# Supplementary material for: ProteinShader: illustrative rendering of macromolecules
Source: BMC Struct Biol. 2009 Mar 30;9:19. doi: 10.1186/1472-6807-9-19 (PMC2672931; doi:10.1186/1472-6807-9-19)
Supplement: Additional file 1 — ProteinShader program without source code. This compressed file contains the complete ProteinShader program including associated libraries, but no source code. A README.txt file gives an overview of the ProteinShader distribution, and the index.html file in the help subdirectory has directions on getting started with the program as well as a set of tutorials. [file 1472-6807-9-19-S1.zip › ProteinShader-beta-0_9_4-binary/help/api/org/proteinshader/structure/enums/class-use/VisibilityEnum.html]

Uses of Class org.proteinshader.structure.enums.VisibilityEnum (ProteinShader API)


|  |  |  |
| --- | --- | --- |
| **Overview** \| **Package** \| **Class** \| **Use** \| **Tree** \| **Deprecated** \| **Index** \| **Help** | |  |
| PREV   NEXT | **FRAMES**    **NO FRAMES**     **All Classes** |  |


---


## **Uses of Class org.proteinshader.structure.enums.VisibilityEnum**

| Packages that use VisibilityEnum | |
| --- | --- |
| **org.proteinshader.gui.components.controlpanel** | Holds the JPanels used in the right-side retractable control panel of the GUI. |
| **org.proteinshader.structure** | Holds the classes that store information from a Protein Data Bank file: Structure, Model, Chain, AminoAcid, Heterogen, Water, Atom, Bond, Helix, BetaStrand, Loop, *etc*. |
| **org.proteinshader.structure.enums** | Holds the enums needed by org.proteinshader.structure and its subpackages. |
| **org.proteinshader.structure.io** | Holds the classes needed for reading Protein Data Bank structure files. |
| **org.proteinshader.structure.visitor** | Holds Visitor classes that know how to traverse the hierarchy of objects held by class Structure. |
| **org.proteinshader.structure.visitor.modifiers** | Holds helper classes for the ModifierVisitor class, which is used to modify Drawable objects (Atoms, Bonds, and Segments). |

Uses of VisibilityEnum in org.proteinshader.gui.components.controlpanel

| Methods in org.proteinshader.gui.components.controlpanel with parameters of type VisibilityEnum | |
| --- | --- |
| `void` | `VisibilityPanel.applyVisibility(VisibilityEnum visibility, float alpha)`             Applies the requested visiblity status and alpha value to whatever item or items are currently selected in the RadioPanel. |
| `abstract  void` | `VisibilityPanel.modifyGlobal(VisibilityEnum visibility, float alpha)`             This helper method for applyVisibility() sets up whatever DrawableModifiers are needed and then calls on the modifyGlobal() method of the radio panel. |
| `void` | `CartoonVisibilityPanel.modifyGlobal(VisibilityEnum visibility, float alpha)`             This helper method for applyVisibility() sets up the the SegmentModifier and then calls on modifyGlobal() method of the radio panel. |
| `void` | `AtomVisibilityPanel.modifyGlobal(VisibilityEnum visibility, float alpha)`             This helper method for applyVisibility() sets up the the AtomModifier and then calls on modifyGlobal() method of the radio panel. |
| `abstract  void` | `VisibilityPanel.modifyHelices(VisibilityEnum visibility, float alpha)`             This helper method for applyVisibility() sets up whatever DrawableModifiers are needed and then calls on the modifyHelices() method of the radio panel. |
| `void` | `CartoonVisibilityPanel.modifyHelices(VisibilityEnum visibility, float alpha)`             This helper method for applyVisibility() sets up a SegmentModifier and then calls on modifyHelices() method of the radio panel. |
| `void` | `AtomVisibilityPanel.modifyHelices(VisibilityEnum visibility, float alpha)`             This helper method for applyVisibility() sets up an AtomModifier and a BondModifier, and then calls on modifyHelices() method of the radio panel. |
| `abstract  void` | `VisibilityPanel.modifyLoops(VisibilityEnum visibility, float alpha)`             This helper method for applyVisibility() sets up whatever DrawableModifiers are needed and then calls on the modifyLoops() method of the radio panel. |
| `void` | `CartoonVisibilityPanel.modifyLoops(VisibilityEnum visibility, float alpha)`             This helper method for applyVisibility() sets up a SegmentModifier and then calls on the modifyLoops() method of the radio panel. |
| `void` | `AtomVisibilityPanel.modifyLoops(VisibilityEnum visibility, float alpha)`             This helper method for applyVisibility() sets up an AtomModifier and then calls on modifyLoops() method of the radio panel. |
| `abstract  void` | `VisibilityPanel.modifySelected(VisibilityEnum visibility, float alpha)`             This helper method for applyVisibility() sets up whatever DrawableModifiers are needed and then calls on the modifySelected() method of the radio panel. |
| `void` | `CartoonVisibilityPanel.modifySelected(VisibilityEnum visibility, float alpha)`             This helper method for applyVisibility() sets up the SegmentModifier and then calls on the modifySelected() method of the radio panel. |
| `void` | `AtomVisibilityPanel.modifySelected(VisibilityEnum visibility, float alpha)`             This helper method for applyVisibility() sets up the AtomModifier and the BondModifier and then calls on the modifySelected() method of the radio panel. |
| `abstract  void` | `VisibilityPanel.modifyStrands(VisibilityEnum visibility, float alpha)`             This helper method for applyVisibility() sets up whatever DrawableModifiers are needed and then calls on the modifyStrands() method of the radio panel. |
| `void` | `CartoonVisibilityPanel.modifyStrands(VisibilityEnum visibility, float alpha)`             This helper method for applyVisibility() sets up a SegmentModifier and then calls on the modifyStrands() method of the radio panel. |
| `void` | `AtomVisibilityPanel.modifyStrands(VisibilityEnum visibility, float alpha)`             This helper method for applyVisibility() sets up an AtomModifier and then calls on modifyStrands() method of the radio panel. |

Uses of VisibilityEnum in org.proteinshader.structure

| Methods in org.proteinshader.structure that return VisibilityEnum | |
| --- | --- |
| `VisibilityEnum` | `Drawable.getVisibility()`             Returns the visibility status of the Drawable object. |

| Methods in org.proteinshader.structure with parameters of type VisibilityEnum | |
| --- | --- |
| `Atom` | `AminoAcid.addNewAtom(int serialNo, AtomEnum type, String atomID, double temperature, int charge, double occupancy, String altLocation, double x, double y, double z, VisibilityEnum visibility)`             Creates a new Atom with the type and Atom ID given as arguments, adds the Atom to the Residue's collection of Atoms, and returns a reference to the new Atom. |
| `Atom` | `Residue.addNewAtom(int serialNo, AtomEnum type, String atomID, double temperature, int charge, double occupancy, String altLocation, double x, double y, double z, VisibilityEnum visibility)`             Creates a new Atom with the type and Atom ID given as arguments, adds the Atom to the Residue's collection of Atoms, and returns a reference to the new Atom. |
| `Atom` | `Structure.addNewAtom(int atomSerialNo, AtomEnum atomType, String atomID, String aminoAcidType, String residueID, String chainID, String modelID, double temperature, int charge, double occupancy, String altLocation, double x, double y, double z, VisibilityEnum visibility)`             Creates a new atom with the atomID given as an argument, adds the new Atom to the requested Model-Chain-AminoAcid, and then returns a reference to the new Atom. |
| `void` | `Atom.addNewBond(Atom dstAtom, BondEnum type, VisibilityEnum visibility)`             Adds a new Bond with this Atom as the source Atom and the Atom given as an argument as the destination Atom. |
| `Atom` | `Structure.addNewHetAtom(int atomSerialNo, AtomEnum atomType, String atomID, String hetName, String residueID, String chainID, String modelID, double temperature, int charge, double occupancy, String altLocation, double x, double y, double z, VisibilityEnum visibility)`             Creates a new atom with the atomID given as an argument, adds the new Atom to the requested Model-Chain-(Heterogen or Water), and then returns a reference to the new Atom. |
| `void` | `Drawable.setVisibility(VisibilityEnum visibility)`             Sets the visibility status of the Drawable object. |

Constructors in org.proteinshader.structure with parameters of type VisibilityEnum

`Atom(int serialNo,
AtomEnum type,
String atomID,
String residueID,
String chainID,
String modelID,
String structureID,
double temperature,
int charge,
double occupancy,
String altLocation,
double x,
double y,
double z,
VisibilityEnum visibility)`
  
          This constructor uses the type argument (an AtomEnum) to set the
default radius and CPK color for the Atom.

`Bond(Atom srcAtom,
Atom dstAtom,
BondEnum type,
VisibilityEnum visibility)`
  
          Constructs a Bond for the source and destination Atoms given as
arguments.

`Drawable(double x,
double y,
double z,
VisibilityEnum visibility,
DrawableEnum drawableType)`
  
          Constructor for use by subclasses.

`Drawable(double x,
double y,
double z,
VisibilityEnum visibility,
DrawableEnum drawableType,
float red,
float green,
float blue,
float alpha)`
  
          Constructor for use by subclasses.

Uses of VisibilityEnum in org.proteinshader.structure.enums

| Methods in org.proteinshader.structure.enums that return VisibilityEnum | |
| --- | --- |
| `static VisibilityEnum` | `VisibilityEnum.valueOf(String name)`             Returns the enum constant of this type with the specified name. |
| `static VisibilityEnum[]` | `VisibilityEnum.values()`             Returns an array containing the constants of this enum type, in the order they're declared. |

Uses of VisibilityEnum in org.proteinshader.structure.io

| Fields in org.proteinshader.structure.io declared as VisibilityEnum | |
| --- | --- |
| `static VisibilityEnum` | `PDBLineParser.ATOM_VISIBILITY`             The default visibilty for Atoms. |
| `static VisibilityEnum` | `PDBLineParser.BOND_VISIBILITY`             The default visibilty for Bonds. |

Uses of VisibilityEnum in org.proteinshader.structure.visitor

| Methods in org.proteinshader.structure.visitor with parameters of type VisibilityEnum | |
| --- | --- |
| `void` | `BondGeneratorVisitor.setAABondVisibility(VisibilityEnum visibility)`             Determines what visibility status the Bond generator will use when generating Bonds for AminoAcids. |
| `void` | `BondGeneratorVisitor.setWaterBondVisibility(VisibilityEnum visibility)`             Determines what visibility status the Bond generator use when generating Bonds for Waters. |

Uses of VisibilityEnum in org.proteinshader.structure.visitor.modifiers

| Methods in org.proteinshader.structure.visitor.modifiers with parameters of type VisibilityEnum | |
| --- | --- |
| `void` | `DrawableModifier.setVisibility(VisibilityEnum visibility)`             Stores a memory that the visibility status needs to be set to the value given as an argument. |

---


|  |  |  |
| --- | --- | --- |
| **Overview** \| **Package** \| **Class** \| **Use** \| **Tree** \| **Deprecated** \| **Index** \| **Help** | |  |
| PREV   NEXT | **FRAMES**    **NO FRAMES**     **All Classes** |  |


---

# *Copyright © 2007-2008*
